# Neuritic complexity of hippocampal neurons depends on WIP‐mediated mTORC1 and Abl family kinases activities

**DOI:** 10.1002/brb3.359

**Published:** 2015-10-03

**Authors:** Ana Franco‐Villanueva, Francisco Wandosell, Inés M. Antón

**Affiliations:** ^1^ Centro Nacional de Biotecnología (CNB‐CSIC)Darwin 3Campus Cantoblanco28049MadridSpain; ^2^ CIBERNED, Centro Investigación Biomédica en Red de Enfermedades NeurodegenerativasMadridSpain; ^3^ Centro de Biología Molecular Severo Ochoa (CBMSO) (CSIC‐UAM)Nicolás Cabrera 1Campus Cantoblanco28049MadridSpain

**Keywords:** Abl, branching, mammalian target of rapamycin complex, neuritogenesis, S6K

## Abstract

**Introduction:**

Neuronal morphogenesis is governed mainly by two interconnected processes, cytoskeletal reorganization, and signal transduction. The actin‐binding molecule WIP (Wiskott‐Aldrich syndrome protein [WASP]‐interacting protein) was identified as a negative regulator of neuritogenesis. Although WIP controls activity of the actin‐nucleation‐promoting factor neural WASP (N‐WASP) during neuritic differentiation, its implication in signal transduction remains unknown.

**Methods:**

Using primary neurons from WIP‐deficient and wild‐type mice we did an immunofluorescence, morphometric, and biochemical analysis of the signaling modified by WIP deficiency.

**Results:**

Here, we describe the WIP contribution to the regulation of neuritic elaboration and ramification through modification in phosphorylation levels of several kinases that participate in the mammalian target of rapamycin complex 1 (mTORC1)‐p70S6K (phosphoprotein 70 ribosomal protein S6 kinase, S6K) intracellular signaling pathway. WIP deficiency induces an increase in the number of neuritic bifurcations and filopodial protrusions in primary embryonic neurons. This phenotype is not due to modifications in the activity of the phosphoinositide 3 kinase (PI3K)‐Akt pathway, but to reduced phosphorylation of the S6K residues Ser^411^ and Thr^389^. The resulting decrease in kinase activity leads to reduced S6 phosphorylation in the absence of WIP. Incubation of control neurons with pharmacological inhibitors of mTORC1 or Abl, two S6K regulators, conferred a morphology resembling that of WIP‐deficient neurons. Moreover, the preferential co‐distribution of phospho‐S6K with polymerized actin is altered in WIP‐deficient neurons.

**Conclusion:**

These experiments identify WIP as a member of a signaling cascade comprised of Abl family kinases, mTORC1 and S6K, which regulates neuron development and specifically, neuritic branching and complexity. Thus, we postulated a new role for WIP protein.

## Introduction

The precise regulation of neuron morphogenesis is a central event in the development of the functional properties of neurons, and thus forms the cellular basis of nervous system function (Kollins and Davenport 2005). Given the complexity of the neuronal network in vivo, cell culture of isolated neurons is used as a model to study the molecular processes that control this sophisticated morphogenesis (Dotti et al. 1988). In this cell system, early postmitotic rounded neurons initiate protrusion of three to five minor neurites; after several hours, one becomes polarized and forms a single axon, whereas the remaining neurites differentiate to dendrites that branch during maturation for correct connectivity and function (da Silva and Dotti 2002). The molecular mechanisms that underlie this morphogenetic process, and specifically the spatial and temporal molecular events that orchestrate the cytoskeletal and signaling changes involved, are not fully understood.

From the onset, extension of the initial neurites is regulated by the cytoskeleton; microtubules (MT) and actin microfilaments (MF) have a key function in neuritic initiation and in the maintenance of neuron morphology and polarity (Black and Baas 1989; Baas 1997; Yin et al. 2008; Arnold 2009). These two cytoskeletal elements have some opposing functions: general MT depolymerization with drugs blocks initial neuritic elongation and growth, whereas MF depolymerization leads to generation of multiple axons (Bradke and Dotti 1999). These data and other reports led to the proposal that MF and MT control neuritic extension, complexity, morphological properties, and subsequent polarization.

In neurons, as in many other cell types, MF dynamics relies mostly on F‐actin polymerization controlled by the conserved Arp2/3 (actin‐related proteins) complex and by nucleation‐promoting factors such as cortactin and neural‐Wiskott Aldrich syndrome protein (N‐WASP) (Pollard et al. 2000; Hering and Sheng 2003; Racz and Weinberg 2004; Tsuchiya et al. 2006; Wegner et al. 2008; Padrick and Rosen 2010). There is, however, much less information about the role of the cortactin‐ and N‐WASP‐binding protein WIP (WASP‐interacting protein) in neurons. In other cell types, WIP binds to actin as well as to N‐WASP and cortactin, which regulate actin nucleation activity at specific sites (Paunola et al. 2002; Anton et al. 2007). We reported WIP expression in adult mouse brain and cultured embryonic cortical and hippocampal neurons; WIP deficiency promotes premature neuritic initiation and dendritic maturation in primary murine neurons without affecting axon complexity or number (Franco et al. 2012). Our results, based on the use of the pharmacological inhibitor wiskostatin, indicate that WIP controls N‐WASP activity in embryonic murine neurons. By controlling activity of an actin‐binding protein and by extension, actin dynamics, WIP thus acts as a negative regulator of soma size as well as of neuritic initiation and maturation in vitro and in vivo.

A number of extracellular signals regulate neuron morphogenesis through various pathways. The IGFR1 (insulin‐like growth factor receptor‐1)‐PI3K‐Akt pathway controls neuritic outgrowth and promotes axonal polarity and dendritic branching (Sosa et al. 2006). Some elements in this pathway, such as Par3/Par6/aPKC, GSK3 (glycogen synthase kinase 3) or the multiprotein complex mammalian target of rapamycin complex 1 (mTORC1) are implicated directly in the generation and/or maintenance of neuronal polarity and complexity (Jaworski et al. 2005; Jiang et al. 2005; Gartner et al. 2006; Jaworski and Sheng 2006). Mammalian Target of Rapamycin (mTORC1and mTORC2) are central mediators of cell growth and of increase in cell mass and size (Laplante and Sabatini 2009). Both mTORC1 and S6K regulate soma size and dendritic arborization in neurons (Kwon et al. 2003). S6K activation is controlled by stepwise phosphorylation of several Serine/Threonine residues located within the autoinhibitory domain, including Ser^411^ (Dufner and Thomas 1999). The phosphorylation at Ser^411^ is required for the subsequent phosphorylation of Thr^389^ site which may allow the full activation of this kinase (Hou et al. 2007). Another signaling family, the Abelson murine leukemia tyrosine kinases (Abl) which consists of Abl and Abl2 (also called Arg), modulate dendrite formation in the developing nervous system through negative regulation of the Enabled protein (de Curtis 2007) or stimulation of Robo/Slit signaling (Bashaw et al. 2000; Wills et al. 2002). Abl‐mediated phosphorylation also controls the stability of dendrite branching in mice, as reduction of kinase levels is accompanied by decreased branching in early adulthood (Moresco et al. 2005).

S6K binds F‐actin directly; this binding is dependent on the S6K phosphorylation level, which promotes actin filament crosslinking and stabilization (Moresco et al. 2005; Ip et al. 2011). The mTORC1‐S6K pathway is involved in actin cytoskeleton reorganization, stimulated by growth factors that induce phosphorylation of focal adhesion proteins (Liu et al. 2008) and promote RhoA expression and activity (Liu et al. 2010). RhoA activity is also modulated by Abl family kinases (Bradley and Koleske 2009).

The WIP contribution to cell signaling is scarcely studied in fibroblasts, lymphocytes, and mast cells (Anton et al. 2007; Noy et al. 2012) and, to our knowledge, has not been addressed in neuron signaling. Here, we analyzed how WIP controls posttranslational modifications that regulate early morphogenesis of primary neurons. Our data indicate that WIP not only has a cytoskeleton‐dependent role, but also governs signaling through mTORC1‐ and Abl family kinases‐dependent modulation of the S6K pathway, which controls neuritic extension and branching.

## Methods

### Mice

WT and WIP^−/−^ SV129/BL6 mice (Anton et al. 2002) were housed in specific pathogen‐free conditions at the animal facility of the Centro de Biología Molecular Severo Ochoa, Madrid, Spain. The colony was maintained by continuous mating of heterozygous females with heterozygous males for more than 25 generations. To obtain control or WIP^−/−^ embryos, we mated homozygous control or WIP^−/−^ pairs of mice. All cell‐related quantification was conducted in a genotype‐blind manner. Mouse handling and all manipulations were carried out in accordance with national and European Community guidelines, and were reviewed and approved by the institutional Animal Care and Use Committee.

### Cell culture and immunofluorescence microscopy

Primary hippocampal or cortical cultures were prepared and processed for immunofluorescence as described (Franco et al. 2012). Briefly, the hippocampus and cortex were obtained from mouse embryos on embryonic day (E)17–18 and rinsed three times in washing solution:Ca^2+^/Mg^2+^‐free HBSS (Hank's balanced salt solution). The hippocampus was digested with 0.25% trypsin (Sigma, St. Louis, MO) and the cortex with 0.25% trypsin plus 1 mg/mL DNase (Roche Diagnostics, Indianapolis, IN), both for 15 min in the washing solution. Tissue was then washed three times in washing solution and dissociated with a fire‐polished Pasteur pipette. Cells were counted, resuspended in plating medium (MEM, 10% horse serum, 0.6% glucose), and plated at a density of 6 × 10^3^ cells/cm^2^ (hippocampal neurons) or 1.5 × 10^5^ cells/cm^2^ (cortical neurons) on poly‐l‐lysine (PLL; Sigma)‐coated coverslips (1 mg/mL). In some experiments, neurons were allowed to adhere to the substrate (1 h) and then incubated with 20 nM Rapamycin (Calbiochem, Darmstad. Germany) or with 1, 3 or 10 μM Imatinib mesylate (Selleck, Houston, TX) both in dimethyl sulfoxide (DMSO). The doses were selected based on published reports using immature primary neuronal cultures (Woodring et al. 2002; Jones et al. 2004; Jaworski et al. 2005; Choi et al. 2008). After 3 h, plating medium was replaced with Neurobasal Medium supplemented with B27 and Glutamax‐I (all from Gibco, Carlsbad, CA). After 24 h of in vitro culture hippocampal cells were fixed in 4% paraformaldehyde in Ca^2+^Mg^2+^‐containing PBS, pH 7.4 (20 min, room temperature), then permeabilized with 0.1% Triton X‐100 and labeled with monoclonal antibody (mAb) to tyrosinated α‐tubulin (TT; 1/400; T9028), TRITC‐phalloidin (1/500; P1951; both from Sigma), and polyclonal anti‐p70S6K‐pThr^389^ (1/60; 9205) or ‐p70S6K antibody (1/60; 9202; both from Cell Signaling Tech, Danvers, MA). The anti‐phosphorylated epitope antibody was incubated in Dako Antibody Diluent with background reducing components (Dako, Glostrup, Denmark) and 5 mM NaF.

Images were recorded digitally on a LSM510 Meta confocal microscope coupled to an inverted Axiovert 200 microscope (both from Zeiss, Oberkochen, Germany) equipped with a CCD camera, and processed with LSM Image Browser software and Adobe Photoshop. Cells showing obvious signs of toxicity, such as neuritic fragmentation/blebbing or vacuoles in the cell body, were excluded from analysis.

### Morphometric analysis

Morphometric measurements of cultured hippocampal neurons were performed with Image J Software (National Institute of Health [NIH], Bethesda, MD). Each experiment was repeated at least three times on independent preparations. Actin and tubulin in neurons were labeled and tubulin distribution was traced for morphometric analysis. Sholl analysis was performed for each traced neuron by automatically calculating the number of neuritic intersections at 20 μm intervals starting from the soma. The total neuritic length and the number of neuritic branch points were counted after 24 h in culture, also calculated as an index of dendritic complexity.

### Fluorescence intensity measurement and multiple dye localization

Fluorescence was measured after applying an inclusive threshold and manually setting threshold limits to prevent pixels from extracellular regions being considered positive. S6K fluorescence intensity per neuron was measured as integrated fluorescence intensity. S6K‐pThr^389^, S6K or F‐actin occupied area per growth cone were determined as percentage of thresholded area per channel over the total growth cone area. For quantitative area measurement of the overlapping region in maximum projection from images of confocal stacks of Alexa488‐anti‐rabbit S6K‐pThr^389^ (green) and TRITC‐phalloidin (red) staining, we first established an intensity threshold for each fluorescent channel. We selected the growth cone F‐actin‐rich area as the one with greater fluorescence intensity and greater pixel density for phalloidin positive signal. The growth cone area comprising the rest of the phalloidin‐associated signal was considered F‐actin sparse. Regarding F‐actin neuritic patches, we first established an intensity threshold for the greater pixel density for phalloidin positive signal in such a way that discrete F‐actin patches could be clearly differentiated from the rest of the neuritic shaft. Then these thresholded areas were selected as F‐actin patches. The adjacent regions with lower signal intensity, F‐actin sparse, were considered as the sides of F‐actin patches and later averaged. The overlap area, that is, the region including integrated intensity (the sum of all intensity values for all pixels in the region) of the green on the red dye, was measured with the Measure Colocalization tool in the selected regions. All measurements were made using Metamorph 7.5.2.0 software (Molecular Devices, Sunnyvale, CA).

### Cell lysates and Western blot

For biochemical analysis, postmitotic cortical neurons were plated on 1 mg/mL PLL‐coated dishes; all times are given as hours post seeding. Neuronal extracts were obtained in lysis buffer (20 mM HEPES pH 7.4, 100 mM NaCl, 5 mM EDTA, 1% Triton X‐100, 100 mM NaF, 1 mM Na_3_VO_4_, complete protease inhibitor cocktail (Roche Diagnostics) and 1 mM okadaic acid) and resolved by 8 or 10% SDS‐PAGE after calculating protein concentration by Bradford analysis (BioRad, Hercules, CA). Proteins were transferred to nitrocellulose filters, blocked and incubated with polyclonal antibodies anti‐Akt pThr^308^ (1/500; 9275), ‐Akt pSer^473^ (1/500; 9271), ‐Akt (1/1000; 9272), ‐GSK3 pSer^21,9^ α,β (1/1000; 9331), S6K pThr^389^ (1/500; 9205), S6K (1/1000; 9202), ‐S6 ribosomal protein pSer^235/236^ (1/500; 2211), ‐p44/42 MAP kinase (1/1000; 9102), ‐p38MAPK (1/1000; 9212) or ‐SAPK/JNK (1/1000; 9252; all from Cell Signaling Tech), S6K pSer^411^ (1/1000; ab47372; Abcam, Cambridge, UK), ‐PDK1 (1 μg/mL; kindly provided by Prof. Dario Alessi, MCR, Dundee, UK), or mAb anti‐GSK3 α, β (1/1000; 44‐610, Biosource, Paisley, UK), ‐GAPDH (1/2500; OBT 1636; AbD Serotec, Oxford, UK), ‐α‐tubulin (1/2500; T9026, Sigma), ‐ERK pThr^202^/Tyr^204^ (1/1000; 4377), ‐p38MAPK pThr^180^/Tyr^182^ (1/1000; 9215) or ‐JNK pThr^183^/Tyr^185^ (1/1000; 4668; all three from Cell Signaling Tech). All anti‐phosphorylated epitope antibodies were incubated in TBS‐Tween with nonfat dry milk and 5 mM NaF.

After incubation with goat anti‐mouse IgG‐HRP (1/5000; sc‐2005), goat anti‐rabbit IgG‐HRP (1/5000; sc‐2004; both from Santa Cruz Biotech, Santa Cruz, CA) or rabbit anti‐goat Ig‐HRP (1/2000; P 0449; Dako Cytomation, Glostrup, Denmark), binding was visualized by ECL (Amersham Biosciences, Piscataway, NJ).

### Quantification of phosphorylation

Densitometric quantification of bands was performed using ImageJ. Data from independent experiments were first normalized to the band of greatest intensity using the same antibody and in the same membrane; the signal was then normalized relative to that of the total protein control.

### Statistical analysis

Data are presented as mean ± standard error of the mean (SEM). Differences between experimental groups were evaluated for significance using Student's *t*‐test or two‐way ANOVA. For all analyses, a minimum value of *P* < 0.05 was considered significant. All statistical analyses were performed using GraphPad Prism version 5.0. (GraphPad Software, Inc., La Jolla, CA).

## Results

### Numbers of neuritic bifurcations and filopodial protrusions increase in the absence of WIP

**Figure 1:**
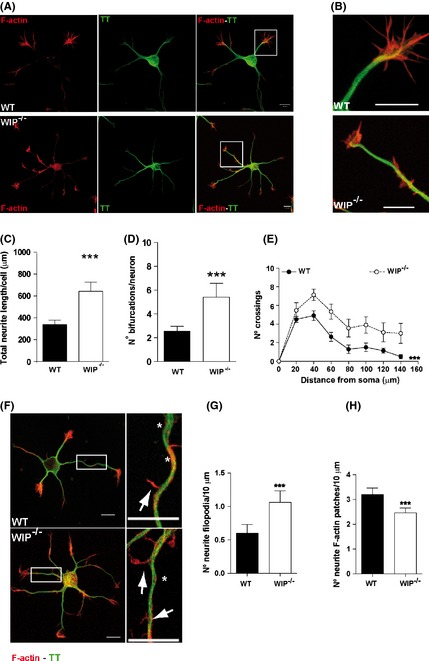
WIP deficiency increases neuritic branching and filopodium formation in dissociated hippocampal murine neurons. (A) Representative confocal images of dissociated embryonic WT and WIP ^−/−^ hippocampal neurons (E18) fixed 24 h post plating and stained for F‐actin (TRITC‐phalloidin, red) and MT (Alexa488‐anti‐tyrosinated tubulin (TT), green). WIP ^−/−^ neuron morphology was more complex than that of WT neurons. Scale bar, 10 μm. (B) High magnification images of the insets in A. Scale bar, 10 μm. (C) Average total neuritic length increased in 24‐h‐cultured WIP ^−/−^ compared to WT hippocampal neurons. The morphology of 30 neurons per genotype was characterized. (D) Average number of bifurcations per neuron increased in WIP ^−/−^compared to WT neurons. (E) Sholl analysis of traced WT and WIP ^−/−^ neurons showing higher number of crossings in WIP ^−/−^ neurons. *n* = 17 neurons analyzed per experimental group, from three independent experiments. ***P < 0.001 (Two‐way ANOVA). (F) Representative confocal images of WT and WIP ^−/−^ neurons obtained and stained as in (A). Right images show higher magnification of the boxed area in left images (scale bar, 10 μm for both). Neuritic filopodia (arrows), neuritic actin patches (asterisks). (G,H) Quantification of neuritic filopodia and actin patches per 10 μm in WT and WIP ^−/−^ neurons. Data for 50 neurons per group in three independent experiments. Student's *t*‐test; ***P < 0.001.

WIP is a negative regulator of early neuritogenesis that controls primary neuritic outgrowth and number (Franco et al. 2012). To determine in detail how WIP affects to this process, we carried out morphometric analyses of primary neuron cultures from control and WIP‐deficient (WIP^−/−^) mice. Cells from trypsin‐dissociated embryonic (E17‐18) hippocampus were seeded onto PLL‐coated coverslips, allowed to differentiate in vitro, fixed, then stained with anti‐tyrosinated α‐tubulin (TT) to label dynamic MT and with TRITC‐phalloidin to label actin MF (see Methods). After 24 h differentiation, neurons reached a morphological stage defined by the presence of tubulin‐rich neurites ending in F‐actin‐rich growth cones (Fig. 1A and B). Morphometric analysis showed that in the absence of WIP, average total neuritic length (tubulin‐positive extensions) per cell was almost twice that of control neurons (641.8 ± 84.9 μm vs. 338.9 ± 39.2 μm; *P* = 0.0009; Fig. 1C). WIP^−/−^ neurons also showed a significant increase in the average number of neuritic branches or bifurcations per neuron (5.4 ± 1.2 vs. 2.5 ± 0.4; *n* = 30; *P* = 0.001; Fig. 1D). Increased complexity was evident when the pattern of branching of the neuritic tree was analyzed quantitatively using Sholl analysis to measure the number of neurites crossing circles at various radial distances from the soma (Sholl 1953). The number of crossings was significantly higher in WIP^−/−^ neurons when compared with WT neurons at 24 h (Fig. 1E). We concluded that the increased total neuritic length observed in the absence of WIP was due to the higher level of neuritic branching, as the average length of each neuritic branch was not modified in WIP^−/−^ neurons (Fig. S1). Therefore, hereafter we analyzed the number of bifurcations per neuron as a measure of neuritic tree complexity. Neuritic sprouting starts at actin‐rich filopodial protrusions (Dent et al. 2007). To test whether the WIP contribution to neuron complexity was linked to this very early event, we quantified the number of neuritic filopodia and F‐actin patches (transient accumulations of F‐actin within the neuritic shaft which serve as precursors for neuritic filopodia [Loudon et al. 2006; Hu et al. 2012; ]) per length unit in WT and WIP^−/−^ neurons. WIP^−/−^ neurons had almost twice as many neuritic filopodia as controls (Fig. 1F and G), whereas the number of neuritic actin patches per length unit decreased significantly in the absence of WIP (Fig. 1H). In addition to confirming our previous report, which showed that neuritogenic activity is accelerated and final complexity enhanced in one‐day‐cultured WIP^−/−^ neurons; these results suggest that early events such as filopodium initiation underlie these processes.

### Akt and GSK3 activities are not substantially modified in WIP^−/−^ neurons

Several signaling pathways govern neuron morphogenesis and polarity, including the crucial IGFR1‐PI3K axis (Sosa et al. 2006). To evaluate WIP influence on signaling pathways that control neuritic elaboration, we analyzed protein levels and/or the phosphorylation state of the PI3K downstream kinases, such as PDK1 (phosphoinositide‐dependent kinase‐1) or Akt.

Akt activity is inferred from phosphorylation levels of its amino acids Thr^308^ and Ser^473^ (Vanhaesebroeck and Alessi 2000), and can also be deduced from the phosphorylation of one of its common substrates, GSK3. Consequently, the phosphorylation level of GSK3‐α Ser^21^ and GSK3‐β Ser^9^ correlates with Akt activity (Cohen and Frame 2001) and PDK1 activity may be deduced from Akt‐Thr^308^ phosphorylation levels (Alessi et al. 1996).

To study Akt activity during neuron development, we allowed cortical neurons from age‐matched WIP^−/−^ and WT embryos to differentiate in vitro for 1–48 h after plating. Cell extracts were prepared and western blot (WB) performed using phosphorylation‐specific antibodies to Akt‐Thr^308^ and ‐Ser^473^, with anti‐Akt antibody as protein loading control.

**Figure 2:**
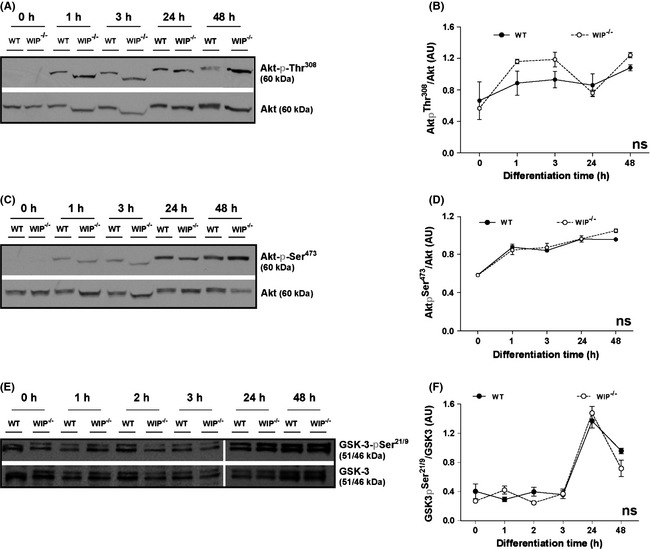
Akt and GSK3 phosphorylation are not modified in WIP ^−/−^ neurons. Soluble extracts were obtained from murine primary cortical neurons from WT or WIP ^−/−^ embryos and maintained in suspension (0) or cultured at high density for 1, 2, 3, 24, or 48 h on PLL‐coated plates. Proteins analyzed in WB (25 μg/lane) were probed with antibodies to phosphoepitopes (A,B) Akt‐pThr^308^, (C,D) Akt‐pSer^473^ or (E,F) GSK3‐pSer^21/9^. Equivalent protein loading was confirmed using anti‐Akt or ‐GSK3 antibodies. Images show one representative experiment. Data show as mean ± SEM; from four independent experiments; ns, not significant (two‐way ANOVA analysis).

In WT neurons, kinetics from the two phospho‐epitopes was similar; both Akt‐pThr^308^ and Akt‐pSer^473^ immunoreactivities increased progressively over a 48‐h period (Fig. 2A–D). We found no significant differences in Akt‐pThr^308^ or Akt‐pSer^473^ phosphorylation values between genotypes in the overall time‐course determined (Fig. 2A–D). To confirm these results, we also analyzed levels of PDK1, the enzyme responsible for Akt‐Thr^308^ phosphorylation, and found no major changes in the amount of total kinase after 24 h of neuron culture, in accordance with data for Akt‐pThr^308^ (Fig. S2).

To confirm that the minor variations in Akt phosphorylation did not influenced Akt kinase activity, we measured phosphorylation of the Akt substrate GSK3. As expected WT neurons showed a slight increase in GSK3‐Ser^21/9^ phosphorylation, evident at 24 and 48 h post plating. Specifically, the increase in GSK3‐Ser^21/9^ phosphorylation observed at 24h post plating is concurrent with the time described for axon initiation in vitro*,* an event dependent on GSK3 activity (Jiang et al. 2005; Garrido et al. 2007; Hur and Zhou 2010). As anticipated from previous results, we found no substantial differences in GSK3‐pSer^21/9^ levels between WIP^−/−^ and WT primary neuron extracts (Fig. 2E–F). The results indicate that, in murine primary neurons, WIP does not contribute notably to signaling pathways involving Akt‐GSK3 phosphorylation and activation.

### S6K activity is reduced in WIP^−/−^ neurons

RheB/mTORC1/S6K is another major PI3K pathway component that regulates neuritic extension and neuron polarity (Jaworski and Sheng 2006; Morita and Sobue 2009). In addition to its role in neuritogenesis, mTORC1 is a general regulator of protein synthesis, cell growth and size in many cell types (Laplante and Sabatini 2009). To infer mTORC1/S6K pathway activity during in vitro neuronal development, we cultured cortical neurons from WIP^−/−^ and WT mice as above and determined the S6K phosphorylation level.

The S6K‐Thr^389^ phosphorylation level is associated with mTORC‐1 activity (reviewed in Ekim et al. 2011; Zoncu et al. 2011) in contrast to S6K‐Ser^411^ phosphorylation which has been reported as mTORC1 independent (Schalm et al. 2005; Hou et al. 2007).

**Figure 3:**
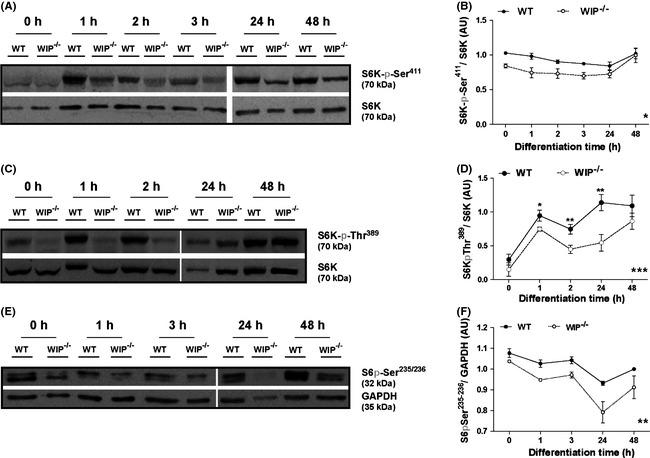
Phosphorylation of S6K and of S6 is reduced in WIP ^−/−^ cells during neuron differentiation. Soluble extracts were obtained from murine primary cortical neurons from WT or WIP ^−/−^ embryos and maintained in suspension or cultured as in Fig. 2. Proteins were analyzed in WB (25 μg/lane) using antibodies to phosphoepitopes (A,B) S6K‐pSer^411^, (C,D) S6K‐pThr^389^, or (E,F) S6‐pSer^235/236^. Equivalent protein loading was confirmed with anti‐S6K or ‐GAPDH antibodies. (B,D,F) Densitometric analysis of the relative amount of each phosphoprotein relative to total protein. Images show a representative experiment. Data show as mean ± SEM; from four independent experiments. Black asterisks over the means refer to the statistical significance of mean differences between the two genotypes for each discrete time point. Gray asterisks placed in the lower right corner of the graphs refer to the statistical significance of differences between the distributions. (B) *P < 0.05 (two‐way ANOVA), (D) *P < 0.05; **P < 0.01 (Student's *t*‐test); ***P < 0.001 (two‐way ANOVA), (F) **P < 0.01 (two‐way ANOVA).

Soluble cell extracts were obtained from primary neurons plated from time 0 to 48 h, and WB performed using phosphorylation‐specific S6K‐Ser^411^ and ‐Thr^389^ antibodies, and anti‐S6K antibody as loading control. A variety of reporters can be used to quantify activity in this pathway; Abl‐mediated Cdk5/p35 activity, detected from the S6K‐pSer^411^ immunoreactivity (Hou et al. 2007), was significantly different between WT and WIP^−/−^ neurons (Fig. 3A and B). The phosphorylation level of S6K‐Thr^389^ and/or that of S6, its main substrate, are commonly measured to determine final activity (Ruvinsky et al. 2005; Ekim et al. 2011; Zoncu et al. 2011). We observed that mTORC1 activity increased at 1 h post plating in control neurons, as inferred from the S6K‐pThr^389^ level (Ekim et al. 2011), whereas S6K‐Thr^389^ phosphorylation was significantly lower in WIP^−/−^ neurons up to 24 h post plating (Fig. 3C and D).

To determine whether these differences effectively influence S6K activity, we used a specific antibody to test phosphorylation at residues Ser^235/236^ of S6, the main S6K substrate (Meyuhas 2008). The temporal pattern of S6 phosphorylation at Ser^235/236^ slowly decrease up to 24 h post platting being more pronounced and statistically lower in WIP^−/−^ neurons compared to the WT kinetics (Fig. 3E and F).

These data correlated with the lower activity inferred from S6K‐Ser^411^ and ‐Thr^389^ phosphorylation (Fig. 3B and D) and confirm reduced S6K activity in WIP^−/−^ neurons.

### The Abl family kinases inhibitor imatinib reduces S6K‐Ser^411^ phosphorylation and increases neuritic complexity in WT neurons

**Figure 4:**
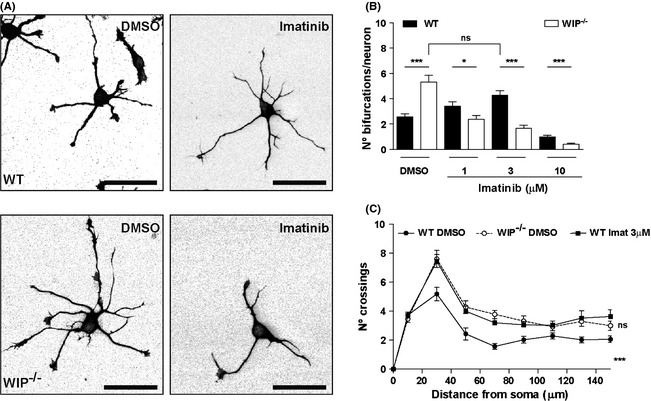
Imatinib inhibition of Abl kinases increases neuritic bifurcations in WT cells, mimicking WIP deficiency. (A) Representative images of dissociated WT and WIP ^−/−^ hippocampal neurons incubated for 24 h with DMSO or 3 μM imatinib, fixed, and stained with anti‐TT mAb. Scale bar, 50 μm. (B) Quantification of experiments in (A). Note the larger number of bifurcations in WIP ^−/−^ neurons in DMSO, which is equivalent to that of WT neurons in 3 μM imatinib. *n* = 150 neurons analyzed per experimental group, from three independent experiments; ns, not significant; *P < 0.05; ***P < 0.001 (Student's *t*‐test). (C) Sholl analysis of traced neurons, after imatinib treatment of WT and WIP ^−/−^ cells. The quantification shows higher number of crossings in WIP ^−/−^ neurons compared to WT cells before treatment. After treatment with imatinib 3 μM, WT neurons show increased branching compared to DMSO‐treated WT neurons. *n* = 30 neurons analyzed per experimental group, from three independent experiments. ***P < 0.001 (WIP ^−/−^ neurons compared to WT cells before treatment); ns, not significant (imatinib‐treated WT neurons compared to DMSO‐treated WIP ^−/−^ cells) (Two‐way ANOVA).

The S6K‐Ser^411^ phosphorylation level is associated with Abl‐mediated Cdk5/p35 activity (Kumar et al. 2000; Hou et al. 2007). To test whether reduced Abl activity is responsible for the differences observed between WIP^−/−^ and WT neurons, we cultured primary neurons from both genotypes alone, with the Abl inhibitor imatinib, or with DMSO. After 24 h, soluble cell extracts were obtained and WB performed using anti‐S6K‐pSer^411^ antibodies to confirm that the pharmacological treatment was effective. Imatinib addition reduced S6K‐pSer^411^ immunoreactivity in WIP^−/−^ and WT neurons without affecting S6K‐pThr^389^ levels (Fig. S3). Abl inhibition in WT neurons by 3 μM imatinib treatment led to S6K‐pSer^411^ phosphorylation levels resembling those of control DMSO‐treated WIP^−/−^ neurons (Fig. S3B), suggesting that WIP deficiency modifies Abl activity during neuronal differentiation. The morphological effects of Abl inhibition were confirmed in imaging experiments; WIP^−/−^ or WT neurons were incubated with imatinib, fixed, and stained with anti‐TT antibody. Morphology analysis of number of bifurcations per neuron showed that neuronal complexity increased in imatinib‐treated WT cells, mimicking that of WIP^−/−^ neurons (Fig. 4A and B), whereas Abl inhibition reduced the number of bifurcations in WIP^−/−^ neurons. Sholl analysis supports these results (Fig. 4C). These data show the requirement of Abl family kinases activity for S6K phosphorylation over early neuron development in vitro and suggest that WIP might negatively regulate neuronal maturation modulating S6K activation by Abl.

### The mTORC1 inhibitor rapamycin modifies neuritic branching

As previously mentioned, S6K‐Thr^389^ is associated with mTORC‐1 activity, whereas S6K‐Ser^411^ appears to be mTORC1‐independent, as it is rapamycin‐insensitive (Schalm et al. 2005; Hou et al. 2007).

To determine whether mTORC1 activity is responsible for the morphological and biochemical differences observed in WIP^−/−^ neurons, we cultured control and WIP^−/−^ cells with rapamycin, with the DMSO vehicle, or untreated. At different times postplating (0, 3, and 24 h), soluble cell extracts were obtained from WIP^−/−^ and WT neurons and WB performed using antibodies specific for S6K‐pThr^389^, S6K‐pSer^411^, and S6‐pSer^235/236^ to verify that rapamycin was inhibiting mTORC1 activity. As expected, rapamycin slightly block S6K‐Ser^411^ phosphorylation (Fig. S4A and B), and as predicted, strongly blocked S6K‐Thr^389^ and S6‐Ser^235/236^ phosphorylation in both neuronal genotypes at 24 h (Fig. S4A, C and D).

**Figure 5:**
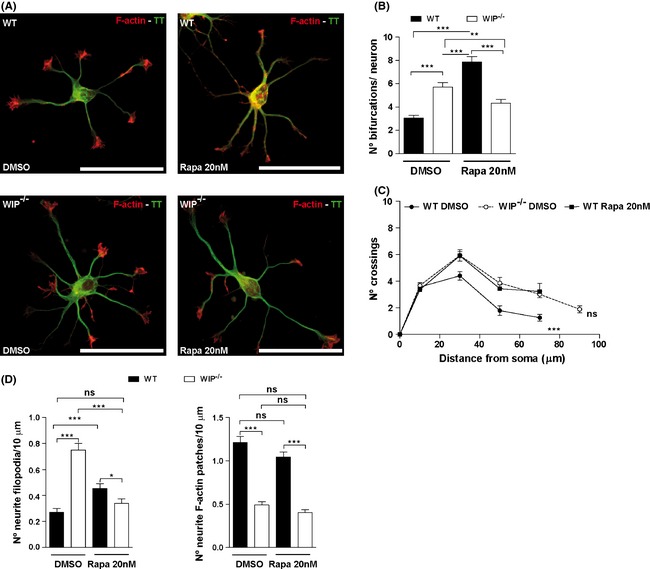
Rapacymin inhibition of mTOR in WT neurons phenotypically mimics WIP ^−/−^ neuritic complexity. Primary neurons from WT or WIP ^−/−^ murine embryos were dissociated and cultured with vehicle (DMSO), or with rapamycin (20 nM). (A) Representative images of rapamycin‐treated neurons fixed at 24 h postplating and co‐labeled with TRITC‐phalloidin (F‐actin, red) and Alexa488‐anti‐ α‐tyrosinated tubulin (TT, green). Scale bar, 50 μm. (B) Average number of bifurcations *per* neuron, alone or rapamycin‐treated. *n* = 100 neurons analyzed per experimental group from two independent experiments. **P < 0.01; ***P < 0.001 (Student's *t*‐test). (C) Sholl analysis of traced neurons, after indicated treatments of WT and WIP ^−/−^ cells. The quantification shows higher number of crossings in WIP ^−/−^ neurons compared to WT cells before treatment. After rapamycin treatment, WT neurons show similar number of crossings that WIP ^−/−^ neurons before treatment. *n* = 30 neurons analyzed per experimental group from three independent experiments. ***P < 0.001 (WIP ^−/−^ neurons compared to WT cells before treatment); ns, not significant (rapamycin‐treated WT neurons compared to DMSO‐treated WIP ^−/−^ cells) (Two‐way ANOVA). (D) Quantification of neuritic filopodia and F‐actin patches per 10 μm in WT and WIP ^−/−^ neurons. Data for 350 filopodia and 355 F‐actin patches, *n* = 40 neurons analyzed per experimental group from three independent experiments. *P < 0.05, ***P < 0.001; ns, not significant (Student's *t*‐test).

In analogous experiments, rapamycin‐treated WIP^−/−^ and WT neurons were fixed and stained with TRITC‐phalloidin and anti‐TT antibody (Fig. 5A). In parallel with reduced S6K‐pThr^389^ levels, the number of bifurcations per neuron and neuritic complexity increased in rapamycin‐treated WT neurons (Fig. 5A–C). Rapamycin treatment of WT neurons mimicked and, indeed, magnified the effect of WIP deficiency by increasing the number of bifurcations per neuron compared to DMSO‐treated WIP^−/−^ neurons. The increase in soma area associated with rapamycin administration in WT neurons confirms the effect on soma area described for WIP deficiency (Franco et al. 2012) (Fig. S5A). The phenotypes induced by mTORC1 inhibition in controls resembled those of WIP^−/−^ neurons (Fig. 5A–C), suggesting that WIP contributes to mTORC1 activity during primary murine neuron differentiation.

In contrast, rapamycin‐treated WIP^−/−^ neurons showed reduced number of bifurcations compared to DMSO‐treated WIP^−/−^ neurons and rapamycin‐treated WT cells (Fig. 5A and B). Our data show that rapamycin induces an increase in the WT neuronal complexity, apparently contradicting published data. Rapamycin has been previously used to define the mTORC1 contribution to neuronal polarization in neurons cultured longer than 3 days (Jaworski et al. 2005; Choi et al. 2008; Li et al. 2008). Thus, we carried out a control experiment to confirm this published data. And certainly (in our experiments) rapamycin treatment of WT neurons for 3 days significantly reduced axonal length (Fig. S5B), supporting the previous reports.

Considering that early neuritogenic events such as filopodium initiation was increased in WIP^−/−^ neurons, we quantified the density of neuritic filopodia and F‐actin patches in rapamycin‐treated WT and WIP^−/−^ neurons to determine whether mTORC1 activity is also responsible for the differences in induction of actin‐based membrane protrusions observed in WIP^−/−^ neurons. Cells were fixed at 24 h post plating and stained with TRITC‐phalloidin and anti‐TT antibody. Vehicle‐treated WIP^−/−^ neurons had almost thrice as many neuritic filopodia as controls (Fig. 5D), whereas the density of neuritic F‐actin patches was decreased compared to control neurons (Fig. 5D). Rapamycin treatment of WT neurons increased the density of neuritic filopodia, whereas it was reduced in rapamycin‐treated WIP^−/−^ neurons. However, the density of F‐actin patches was not affected by rapamycin treatment in either genotype. In addition to being consistent with the effect of rapamycin on neuritic branching and complexity, these results suggest that WIP participates in mTORC1 activity over early protrusive events but not in the formation of its precursors, F‐actin patches.

### S6K‐pThr^389^ is lower in the cell body and neuritic shaft in WIP^−/−^ than in WT neurons

**Figure 6:**
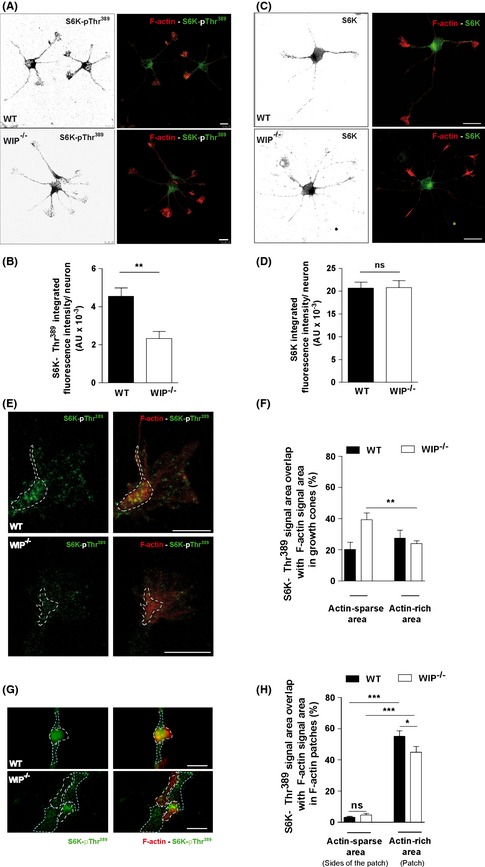
Subcellular S6K‐pThr^389^ distribution is modified in WIP ^−/−^ neurons. Representative images of hippocampal neurons from WT and WIP ^−/−^ murine embryos seeded on PLL‐coated coverslips (24 h), fixed and stained with TRITC‐phalloidin (F‐actin, red) and Alexa488‐anti‐S6K‐pThr^389^ (green) (A) or Alexa488‐anti‐S6K (green) (C). Scale bar, 10 μm for both. Quantification of integrated fluorescence intensity of S6K‐pThr^389^ (B) and anti‐S6K (D) for control and WIP ^−/−^ neurons. (E) Representative images of neuron growth cones obtained following the experimental design described for Fig. 5A and C. Scale bar, 5 μm. (F) Quantification of the spatial overlap of Alexa488‐anti‐S6K‐pThr^389^ signal on TRITC‐phalloidin signal in actin‐sparse or ‐rich (dotted outline) areas. *n *= 12 growth cones per genotype, from two independent experiments. Student's *t*‐test; **P < 0.01. (G) Representative images of neuritic F‐actin patches acquired following the experimental design described for Fig. 5A and C. Scale bar, 1 μm. (H) Quantification of the spatial overlap of Alexa488‐anti‐S6K‐pThr^389^ signal on TRITC‐phalloidin signal in actin‐sparse (light blue dotted outline) or ‐rich (white dotted outline) areas. *n *= 60 F‐actin patches or average of either side of them per genotype, from two independent experiments. *P < 0.05; ***P < 0.001 (Student's *t*‐test).

S6K‐pThr^389^ is found in dendrites and soma of mature neurons (Cammalleri et al. 2003). To determine if its localization is similar in immature neurons, and whether the biochemical differences observed are region specific, we cultured primary hippocampal neurons from WT and WIP^−/−^ mice for 24 h, followed by fixing and staining with TRITC‐phalloidin and anti‐S6K and ‐S6K‐pThr^389^ antibodies. In WT neurons, both total S6K and S6K‐pThr^389^ occupied the soma and neurites, including tips of all extensions; total S6K also showed more apparent perinuclear staining (Fig. 6A and C). Overall S6K‐pThr^389^ fluorescence intensity was lower in WIP^−/−^ neurons (Fig. 6A and B), whereas there was no difference in total S6K fluorescence intensity between genotypes (Fig. 6C and D). We therefore tested whether this reduction was greater in actin‐rich areas, where S6K‐pThr^389^ concentrates in tumor cells (Ip et al. 2011). We focused on growth cones and in neuritic actin patches, the main actin‐rich structures in developing neurons.

The fluorescence intensity and the growth cone area occupied by S6K‐pThr^389^, S6K, or by F‐actin were similar in both genotypes (Fig. S6). However, in WIP^−/−^ neuron growth cones, specific immunofluorescence analysis showed preferential distribution of S6K‐pThr^389^ in regions not enriched in F‐actin (Fig. 6E and F), in contrast to the more uniform S6K‐pThr^389^ distribution in control neuron growth cones (Fig. 6E and F).

Finally, we performed a similar quantification to analyze the distribution of S6K‐pThr^389^ relative to F‐actin patches that spread over neuritic shafts and precede the formation of neuritic filopodia, essential for neuritic sprouting. In both genotypes specific immunofluorescence analysis showed preferential distribution of S6K‐pThr^389^ in F‐actin patches compared with surrounding areas. In contrast, the levels of S6K‐pThr^389^ are decreased in F‐actin patches of WIP^−/−^ neurons compared to control cells (Fig. 6G and H). These findings indicate that WIP is a regulator of S6K‐pThr^389^ levels and spatial distribution, linked to kinase proximity to F‐actin.

## Discussion

Proper neuritogenesis shapes the integration of neurons into functional circuits. Traditionally, research on intracellular signaling has focused on axon formation as the earliest stage of neuritogenesis. However, neuritic initiation is the foremost event of neuronal morphogenesis (Flynn 2013). Here, we show that WIP participates in the establishment of neuritic complexity during early neuritic initiation modulating the activity of the Abl‐mTORC1‐S6K pathway. Moreover, in the absence of WIP the density of neuritic filopodia is increased and the levels of active S6K are decreased in MF‐rich area of the growth cones. Our findings identify WIP as a key modulator of signaling events that critically determine actin‐dependent neuritic outgrowth.

**Scheme 1:**
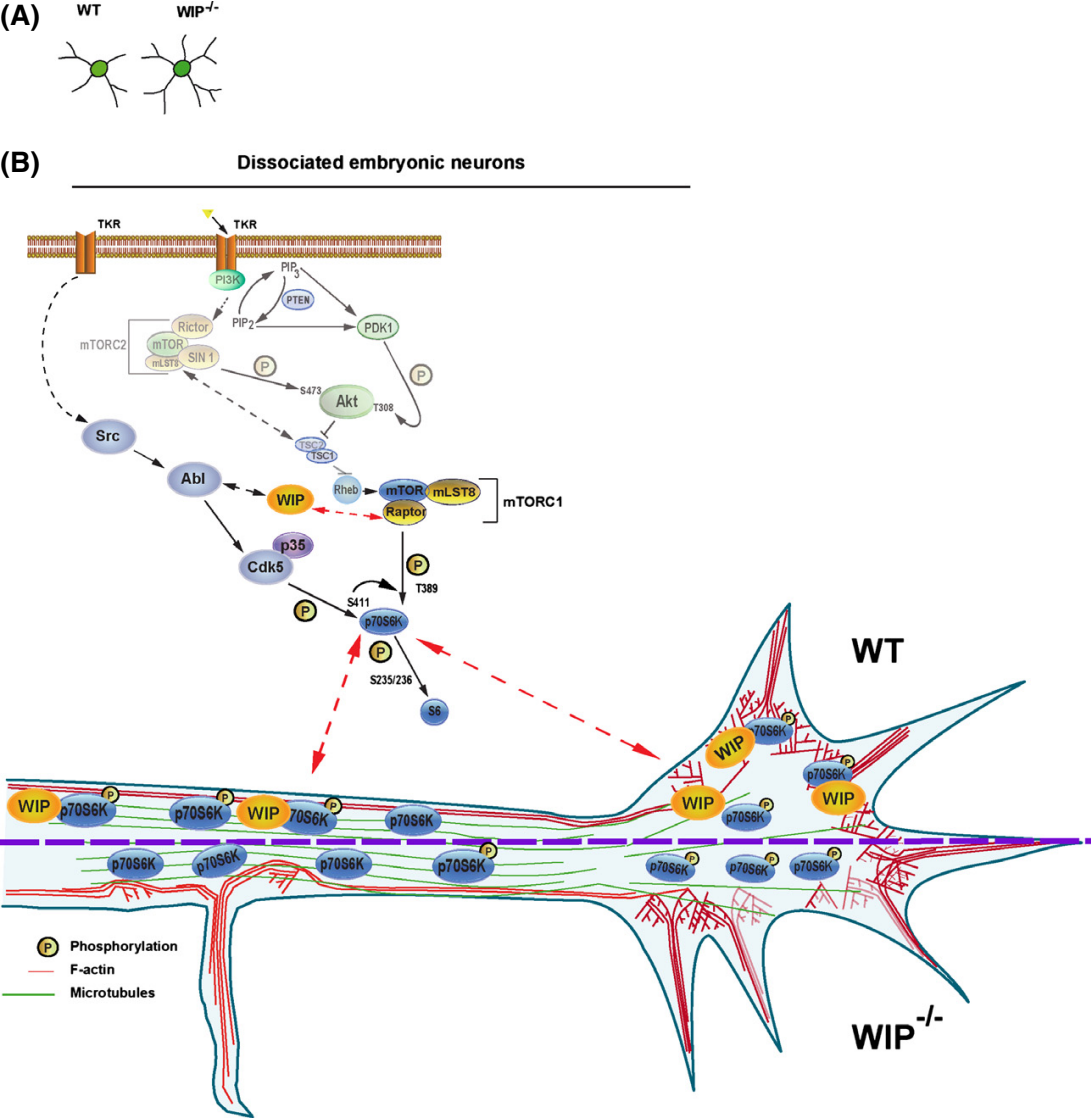
WIP controls neuritic branching through the modulation of the activity level of the Abl‐mTORC1‐S6K pathway. (A) Archetypical morphology of WT and WIP ^−/−^ neurons at 24 h post plating derived from our quantitative IF and biochemical data. (B) Our data permit to postulate that in a model system of dissociated embryonic neurons, WIP determines the level of neuritic branching via the positive regulation of the mTORC1‐S6K pathway activity for the initial stages of neuronal development. The effect in two phospho‐epitopes of S6K allows hypothesizing that, in addition to mTORC1 activation (S6K‐pThr^389^), Abl may be implicated in this initial regulation through Cdk5‐p35 kinase complex (S6K‐pSer^411^). As the levels of active S6K and F‐actin are equivalent in the growth cones of both genotypes, WIP might play a potential role as a scaffold protein tethering S6K‐pThr^389^ to MF in actin‐rich areas. Moreover, WIP ^−/−^ neurons present reduced levels of S6K‐pThr^389^ over the neurites and increased density of neuritic filopodia compared to controls. These data lead us to propose that WIP and active S6K may act as negative regulators of the F‐actin protrusive activity along the neuritic shaft.

Rescue experiments (Franco et al. 2012), support the implication of WIP in the phenotype described above. WIP^−/−^ neurons were nucleofected with a lentiviral vector coding for control GFP or WIP‐GFP. Only the expression of WIP‐GFP in WIP^−/−^ neurons reversed the average number of neuritic bifurcations per neuron at 24 h after plating (Franco et al. 2012). These data show that the level of neuritic branching is determined by WIP. In early neuritogenesis, WIP controls N‐WASP activity by holding N‐WASP in its autoinhibited state (Franco et al. 2012). Moreover, these new data support that WIP also regulates early neuritogenesis by modulating the activity of Abl‐mTORC1‐S6K pathway in immature neurons. WIP interacts through its proline‐rich domains with proteins that contain SH3‐domains, involved in many intracelullar signaling pathways such as Nck, Grb2, and cortactin, and regulates other cascades including MAPK (mitogen‐activated protein kinase) or nonreceptor tyrosine kinases (Anton et al. 2007; Padrick and Rosen 2010). As MAPK phosphorylation levels depend on WIP expression in murine fibroblast (Lanzardo et al. 2007), our initial analysis focused on MAPK pathways signaling. When we analyzed WIP^−/−^ neurons, we found no significant changes in the phosphorylation levels of these stress‐mediated kinases (including ERK, p38 and JNK), as the minor variations observed between genotypes for specific time points were not consistent among independent experiments (Fig. S7). These data suggest that inhibiting mTORC1 activity in WIP^−/−^ neurons is not a side effect of potential cell stress caused by WIP deficiency. We then studied the PI3K‐Akt‐GSK3 pathway, which plays a central role in neuronal morphogenesis (Jaworski et al. 2005; Jaworski and Sheng 2006). In WIP^−/−^ neurons, activity of this pathway was not statistically modified in the initial steps of neuritic extension and axonal elongation, as determined by Akt activation inferred from phosphorylation levels in its residues Thr^308^ and Ser^473^ and the residues Ser^21/9^ of its substrate GSK3. GSK3 inhibition by Akt is necessary exclusively for the development of axons in primary neurons (Jiang et al. 2005; Garrido et al. 2007; Hur and Zhou 2010). Our results agree with these previous works since WIP is only involved in the development of immature neurites and mature dendrites without affecting axonal number or morphometry (Franco et al. 2012) (Scheme 1A).

Abl, mTORC1 and S6K are other key regulators of neuronal morphogenesis (Moresco and Koleske 2003; Jones et al. 2004; Jaworski and Sheng 2006). Our findings suggest that WIP modifies the capacity of Abl family kinases and of mTORC1 to phosphorylate S6K, as both phosphorylations mediated by those proteins in the residues Ser^411^ and Thr^389^ of S6K, respectively, are reduced in the WIP^−/−^ neurons compared to controls. WIP sequence contains 18 likely Abl interaction sites (http://scansite.mit.edu/). Considering the higher abundance of Abl as compared to Arg during brain development (Moresco 2000) and that Abl is the only kinase that can be related to the phosphorylation of the S6K‐Ser^411^(Zukerberg et al. 2000; Markova et al. 2010), we propose Abl as the most probable candidate for the interaction with WIP between the Abl family members. However, the possibility that Arg might interact with WIP cannot be excluded. Some restrictions of our experimental model make difficult the detection of a protein–protein association as a direct link between WIP and Abl, mTOR, or S6K. First, WIP and Abl are expressed at very low levels at the studied stage of neuronal development (Perez de Arce et al. 2010; Franco et al. 2012). Second, the interaction between WIP, Abl, mTOR, or S6K might be transient or spatially restricted to growth cones or filopodia, as early neuritogenesis relies on highly dynamic and localized molecular events (de Curtis 2007).

The Abl family plays a regulatory role in morphogenetic events including F‐actin microspike assembly stimulation (Woodring et al. 2002). Several studies support a link between Abl and S6K: Cdk5 reaches maximum activation in response to Abl‐mediated phosphorylation (Zukerberg et al. 2000) and then, the Cdk5‐p35 kinase complex phosphorylates S6K at Ser^411^, allowing mTORC1‐mediated S6K activation in the nervous system (Hou et al. 2007). Furthermore, Bcr‐Abl activates the mTORC1‐S6K pathway independently of Akt in diverse cell lines (Markova et al. 2010). Our results correlate well with these data, as Abl family kinases inhibition with imatinib was followed by a decrease in S6K‐Ser^411^ phosphorylation in WT neurons. Taken together, we propose that Abl could mediate a yet‐unidentified connection between WIP, S6K and F‐actin reorganization during neuritogenesis (Scheme 1B).

Our results indicate that S6K activity was significantly reduced in WIP^−/−^ neurons, as inferred from its phosphorylation level (S6K‐Ser^411^ and S6K‐Thr^389^) or determined directly from S6 phosphorylation levels. To analyze the role of the mTORC1‐S6K pathway (Laplante and Sabatini 2009) in neuritic initiation we used the mTORC1 inhibitor rapamycin, which has been used in previous studies mostly to define the contribution of mTORC1 to neuronal polarization in neurons cultured for more than 3 days (Jaworski et al. 2005; Choi et al. 2008; Li et al. 2008). Nonetheless, few data exist regarding the effect of rapamycin in early neuritogenesis. Our experiments using inhibitors imply that the activity of both kinases is necessary for proper neuron morphology in early stages of development. We have shown that inhibiting either of the two kinases, mTORC1 or Abl family in WT neurons generates a more complex morphology in differentiating WT neurons – with highly branched and extended neurites – that mimics the WIP^−/−^ phenotype. Moreover, rapamycin treatment also induces an increase in density of neuritic filopodia in WT neurons, suggesting that mTORC1 is involved in the very first events associated to neurite initiation and branching. With the exception of a previous work in which mTOR and S6K activity was also inversely correlated with the level of neuritic complexity (Guo et al. 2011), the relationship observed between the activity of these pathways and the level of cellular morphological complexity differs from most of published data. These differences may be due to the studied stage of neuron development, as most of the published experiments have been carried out with more mature neurons than those studied in this work (Woodring et al. 2002; Jones et al. 2004; Jaworski et al. 2005; Choi et al. 2008). Neuritogenesis and axonogenesis use the same basic machinery yet in several different ways (Flynn 2013). Thus, each stage of neuronal maturation requires specific changes in the activity of a given signaling pathway (Sala et al. 2000; Sava et al. 2012). In contrast to WT neurons, imatinib or rapamycin‐treated WIP^−/−^ neurons undergo simplification of neuritic complexity and reduction of neuritic filopodia density. This discrepancy might be explained by the dose–response phenomenon termed hormesis, which is characterized by low‐dose stimulation and high‐dose inhibition (Calabrese et al. 2012), as our data are consistent with the criteria established for hormetic phenomena (Calabrese 2013). Namely: quantitative features of the dose–response relationship, statistical significance, replication of findings, signal transduction pathway‐based mechanism, and simulation studies using pathway inhibitors. Hormesis may reflect the biological plasticity that arises through compensatory processes when homeostasis is altered, as it is the case of full knockout mice. Hormetic response pathways involve deacetylases or kinases (Mattson 2008), including the mTOR pathway and c‐Abl (Blagosklonny 2011). In our system, a partial inhibition of both pathways might be required to generate the highest level of neuritic branching in immature neurons, whereas a deeper or total inhibition seems to simplify the neuritic tree.

Our findings thus indicate that WIP ultimately affects S6K activity (Scheme 1A). S6K colocalizes with F‐actin at the leading edge of motile cells (Berven et al. 2004; Ip et al. 2011), therefore we analyzed the distribution of this kinase at the actin‐rich leading edges of the neurites, the growth cones. The signal intensities and areas *per* growth cone of S6K‐pThr^389^, S6K and F‐actin were comparable in WT and WIP^−/−^ neurons (Fig. S6). However, when we examine the localization of active S6K relative to F‐actin, we found that S6K‐pThr^389^ is mainly concentrated in F‐actin sparse regions in the absence of WIP, in contrast to the more uniform S6K‐pThr^389^ distribution observed in growth cones of control neurons. In ovarian cancer cells, phosphorylation of S6K‐pThr^389^ promotes S6K binding to F‐actin (Ip et al. 2011). As the levels of active S6K and F‐actin are equivalent in the growth cones of both genotypes, WIP might play a potential role as a direct or indirect scaffold protein tethering S6K‐pThr^389^ to MF in actin‐rich areas. This hypothesis could explain why S6K‐pThr^389^ is preferentially localized in F‐actin sparse areas in the absence of WIP, despite that the phosphorylation on this residue promotes the binding between F‐actin and S6K.

In addition to growth cones, we have also detected actin‐rich structures along the neurites. Here we show that the density of neuritic filopodia increases while the density of neuritic F‐actin patches decreases in WIP^−/−^ neurons compared to controls. Since most of the filopodia emerge from F‐actin patches, but only a fraction of patches develops to filopodia (Loudon et al. 2006), our data suggest that the absence of WIP increases F‐actin patches conversion to neuritic filopodia along the neuritic shaft. Filopodia are indispensable for neuritic initiation (Dent et al. 2007; Flynn 2013). The actin‐based filopodia first protrude, then MT follow the lead of the advancing filopodia that finally become elongating neurites. A similar process mediates neuritic branching (Dailey and Smith 1996; Lalli and Hall 2005; Flynn et al. 2009), which is also increased in the absence of WIP as we describe in this study. F‐actin must undergo local de‐polymerization triggered by extracellular signals to allow cortical MF rearrangements and MT penetration (Luo 2002; da Silva and Dotti 2002). WIP inhibits F‐actin de‐polymerization (Martinez‐Quiles et al. 2001), so its absence might favor cortical cytoskeleton reorganization, required for the formation of neuritic filopodia and neuritic branches. S6K‐pThr^389^ might be another key player involved in the remodeling of cortical actin, given its localization over neuritic shafts (data presented in this study and reported by Cammalleri et al. 2003) and its ability to bind and stabilize F‐actin (Ip et al. 2011). As we described here, WIP^−/−^ neurons present reduced levels of S6K‐pThr^389^ over the neurites and in neuritic F‐actin patches compared to controls. These data lead us to propose that the reduction of this F‐actin stabilizing protein along the neuritic shaft, and specifically in F‐actin patches, might contribute to destabilize cortical F‐actin and potentially to enhance cortical MF rearrangements essential for filopodium protrusion and neuritic branching.

In summary, this study emphasizes the importance of WIP as an essential cytoskeletal and signaling negative regulator in controlling events that underlie neuronal development. Specifically, we found that WIP and the Abl family kinases‐mTORC1‐S6K pathway are functionally involved in shaping neuritic initiation, the first but least studied stage of neuronal development. Here, we demonstrate that WIP regulates neuritogenesis and neuritic branching controlling Abl‐mTORC1‐S6K activity and the localization of the latter relative to F‐actin‐rich structures. Our study invites future research on the molecular mechanisms underlying these effects. Dendrite development is intimately linked to the emergence of behavioral symptoms in several mental disorders such as schizophrenia and autism. Determining the contribution of WIP to the mechanisms that control dendrite morphogenesis and/or maintenance will not only improve our understanding of nervous system function, but could also help to identify the underlying causes of some of these neurodevelopmental conditions.

## Competing Interests

The authors declare that they have no competing interests.

## Sources of Financial Support

A. Franco‐Villanueva received an FPU fellowship from the Spanish Ministry of Science and Innovation (MICINN). This work was supported by grants from the MICINN: Plan Nacional of the Dirección General de Ciencia y Tecnología (BFU2010‐21374/BMC to I. M Antón and SAF2012‐39148‐C03‐01 to F. Wandosell), the European Union (EU‐FP7‐2009‐CP‐IP 212043‐2–NAD) to F. Wandosell, the Instituto de Salud Carlos III Centro de Investigación Biomédica en Red sobre Enfermedades Neurodegenerativas (CIBERNED), and by an institutional grant from the Fundación Ramón Areces to CBMSO.

## Supporting information


**Figure S1.** Neuritic length per branch order is not modified in 24‐h‐cultured WIP^−/−^ neurons.

**Figure S2.** PDK1 levels are similar in WT and WIP^−/−^ neurons 24 h post plating.

**Figure S3.** Imatinib inhibition of Abl kinases decreases S6K phosphorylation.

**Figure S4.** Rapacymin inhibition of mTOR decreases S6K phosphorylation.

**Figure S5.** Rapamycin inhibition of mTOR differentially modifies soma area and reduces axon length in WT.

**Figure S6.** Fluorescence intensity and area occupied by S6K‐pThr^389^, S6K or by F‐actin are equivalent in WT and WIP^−/−^ growth cones at 24 h after plating.

**Figure S7.** ERK, p38, or JNK phosphorylation levels are similar in cultured WT and IP^−/−^ neurons in the first 48 h post plating.
