# Supplementary figures and images for: Neuritic complexity of hippocampal neurons depends on WIP‐mediated mTORC1 and Abl family kinases activities
Source: Brain Behav. 2015 Oct 3;5(11):e00359. doi: 10.1002/brb3.359 (PMC4667760; doi:10.1002/brb3.359)

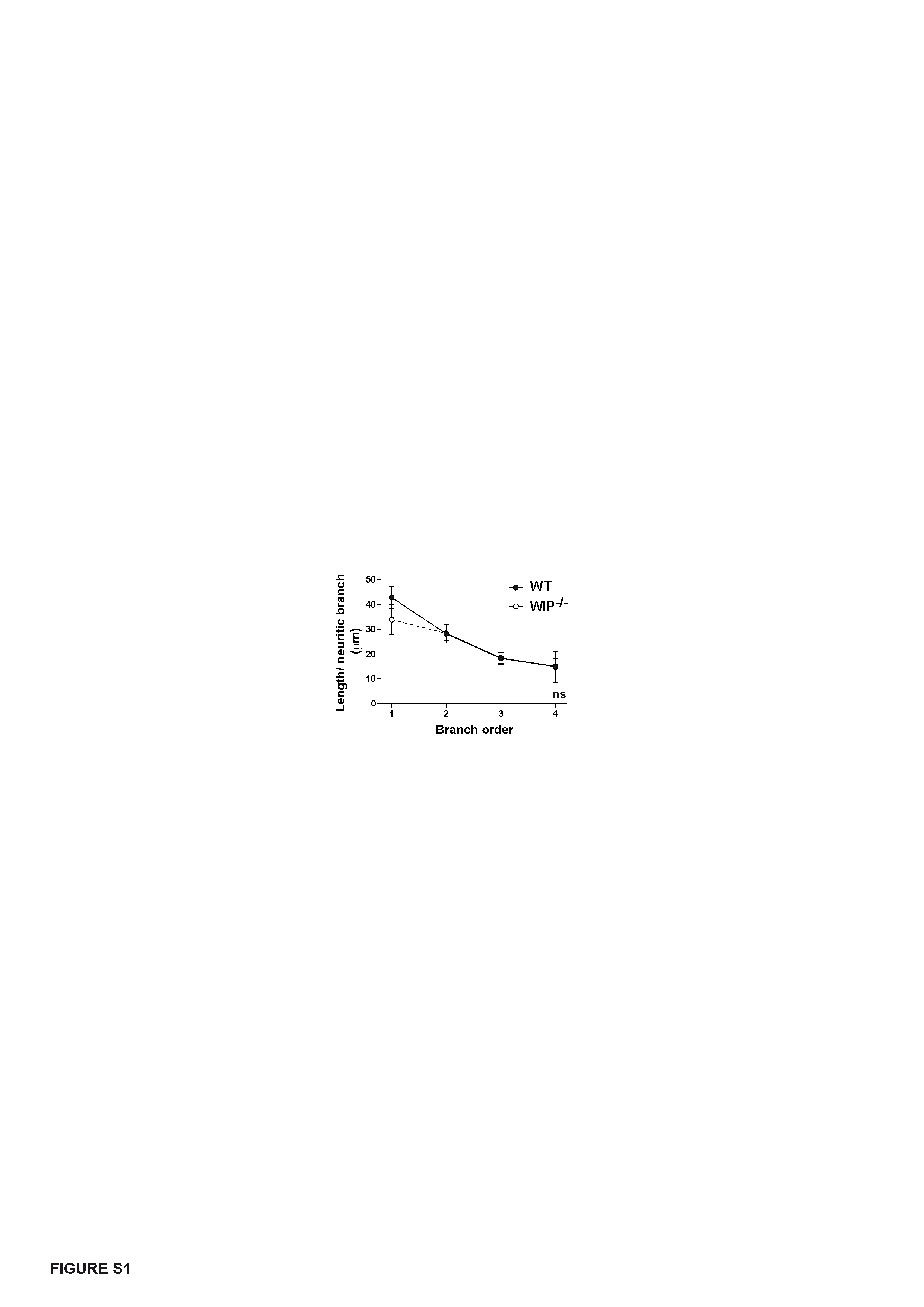

Supplement: Supplementary file 1 — Figure S1. Neuritic length per branch order is not modified in 24‐h‐cultured WIP−/− neurons. [file BRB3-5-e00359-s001.tif]

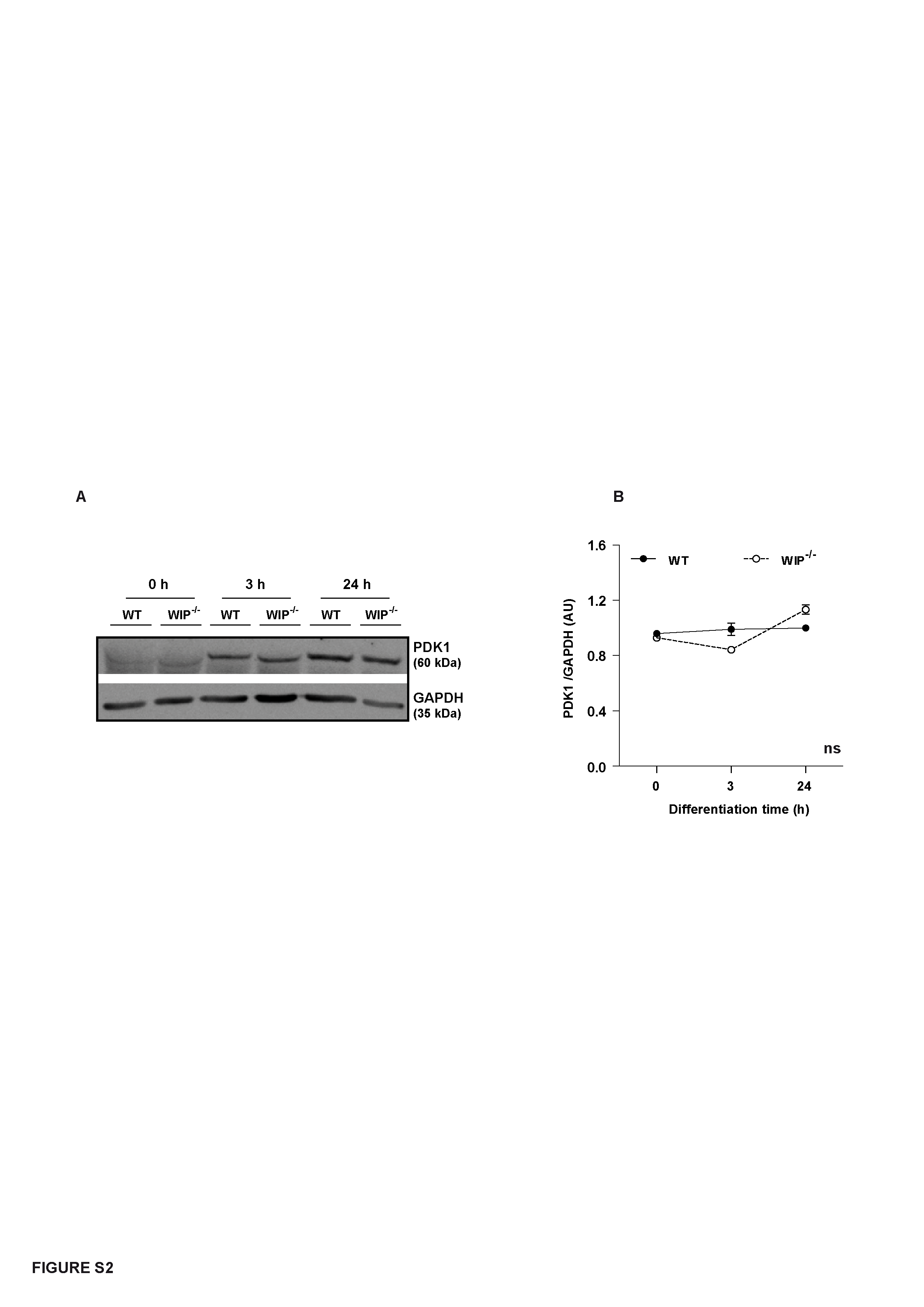

Supplement: Supplementary file 2 — Figure S2. PDK1 levels are similar in WT and WIP−/− neurons 24 h post plating. [file BRB3-5-e00359-s002.tif]

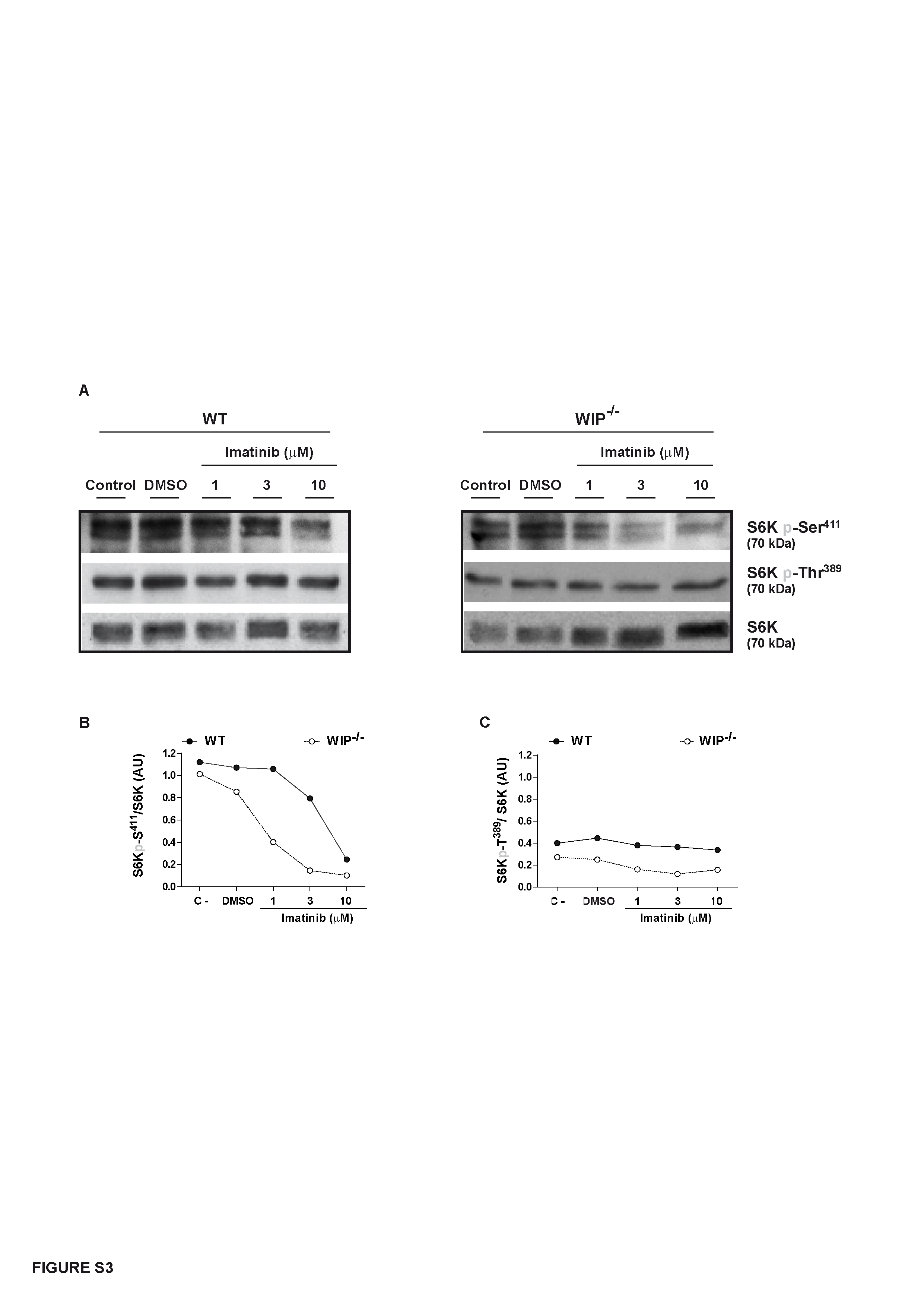

Supplement: Supplementary file 3 — Figure S3. Imatinib inhibition of Abl kinases decreases S6K phosphorylation. [file BRB3-5-e00359-s003.tif]

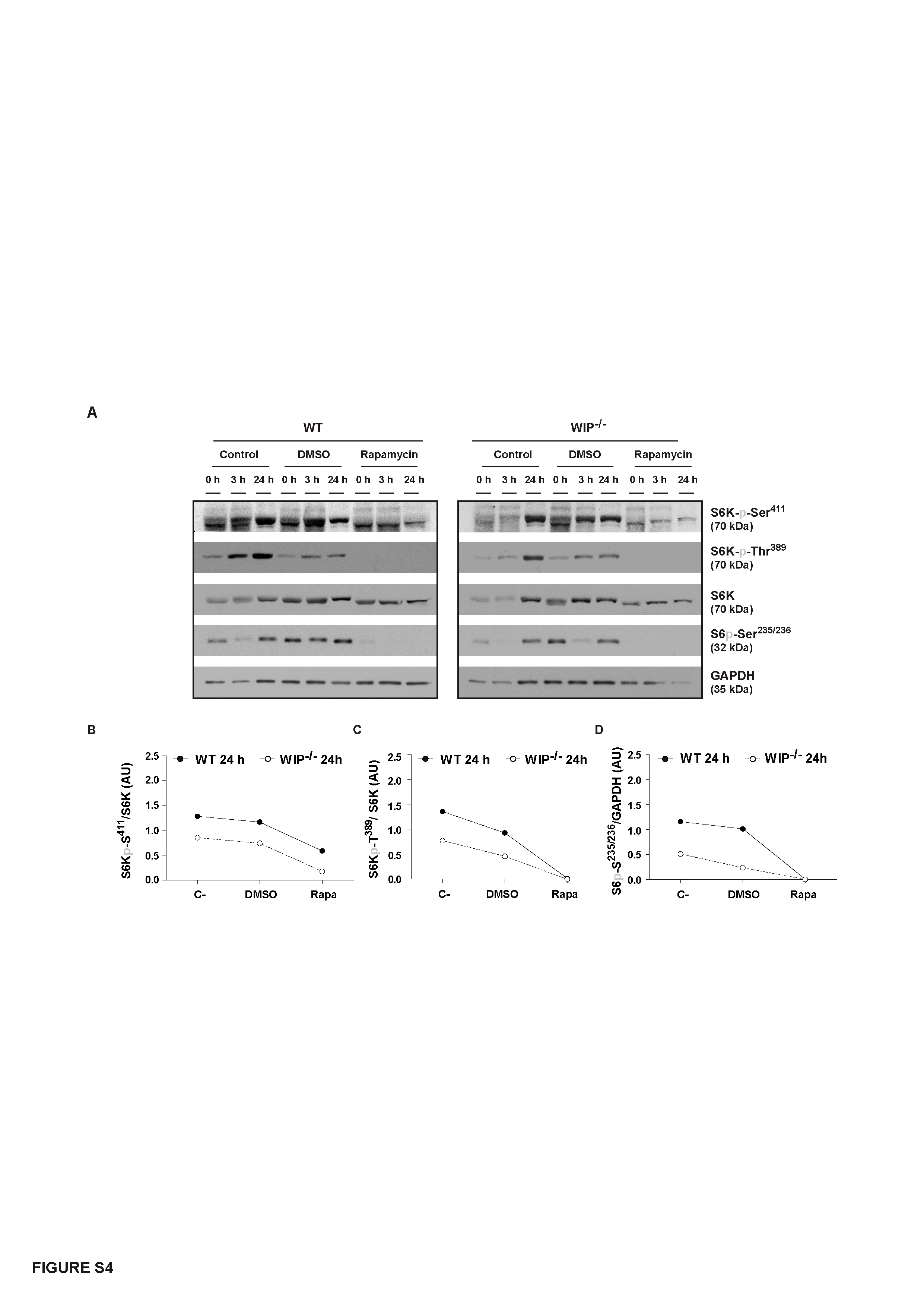

Supplement: Supplementary file 4 — Figure S4. Rapacymin inhibition of mTOR decreases S6K phosphorylation. [file BRB3-5-e00359-s004.tif]

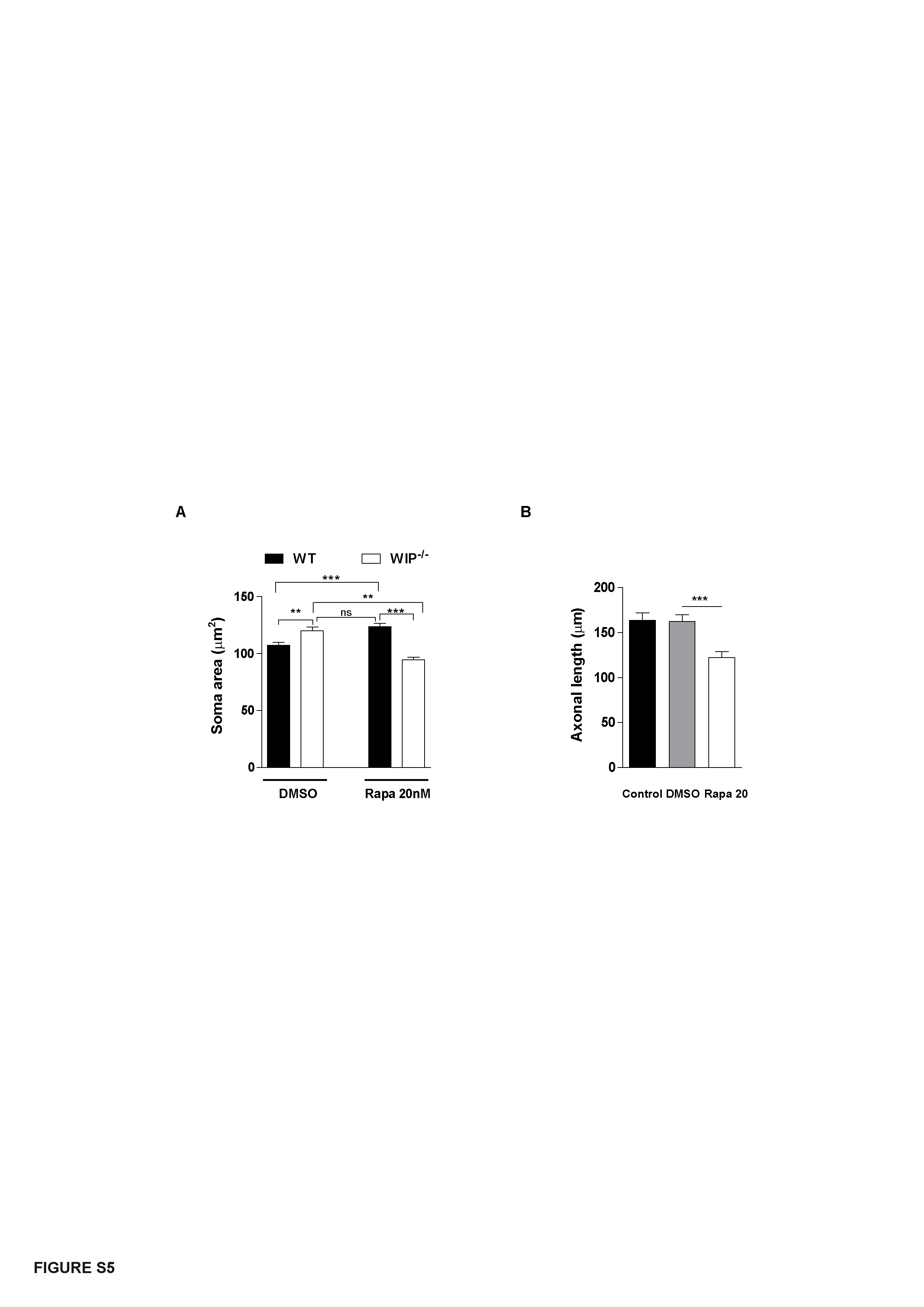

Supplement: Supplementary file 5 — Figure S5. Rapamycin inhibition of mTOR differentially modifies soma area and reduces axon length in WT. [file BRB3-5-e00359-s005.tif]

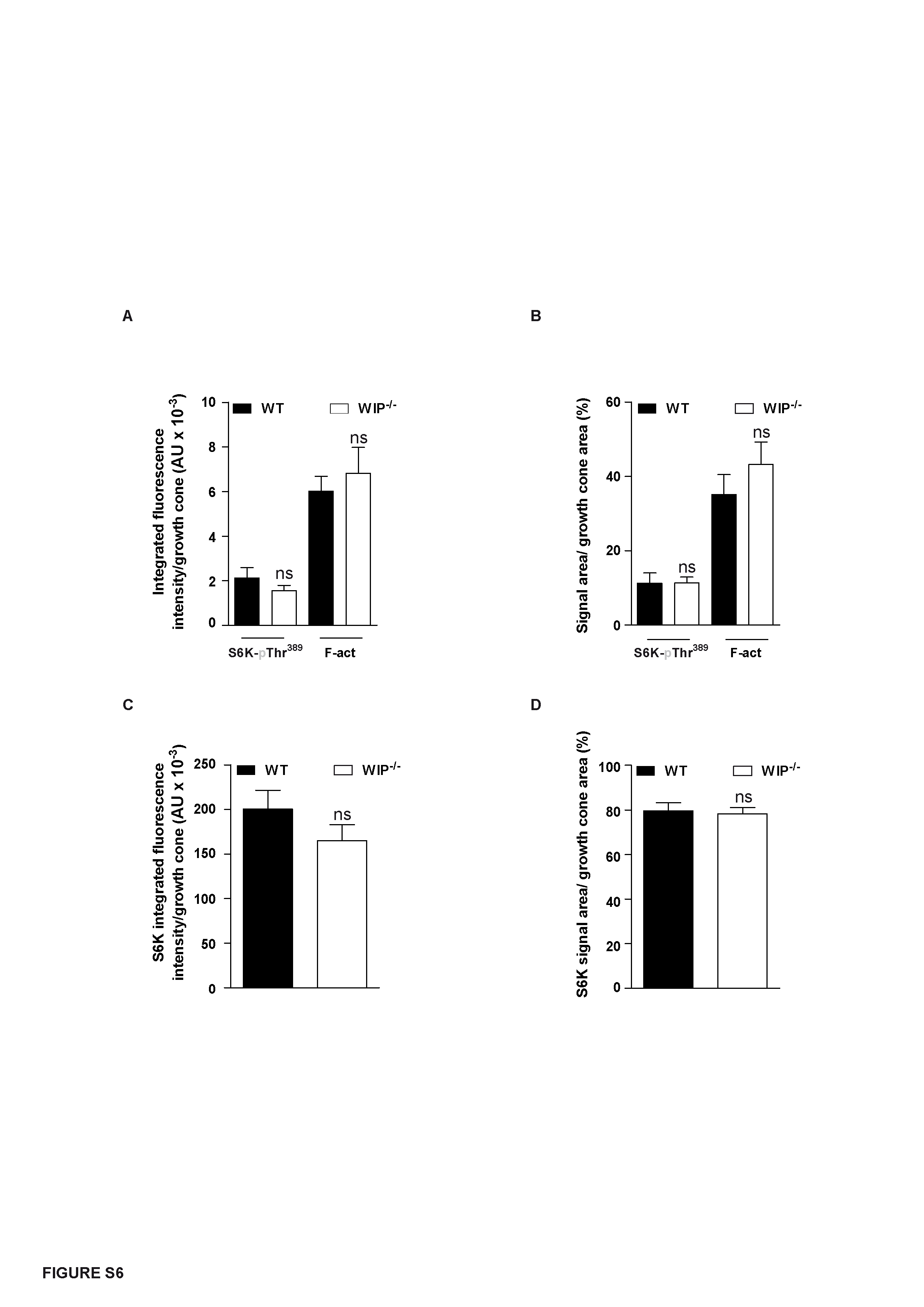

Supplement: Supplementary file 6 — Figure S6. Fluorescence intensity and area occupied by S6K‐pThr389, S6K or by F‐actin are equivalent in WT and WIP−/− growth cones at 24 h after plating. [file BRB3-5-e00359-s006.tif]

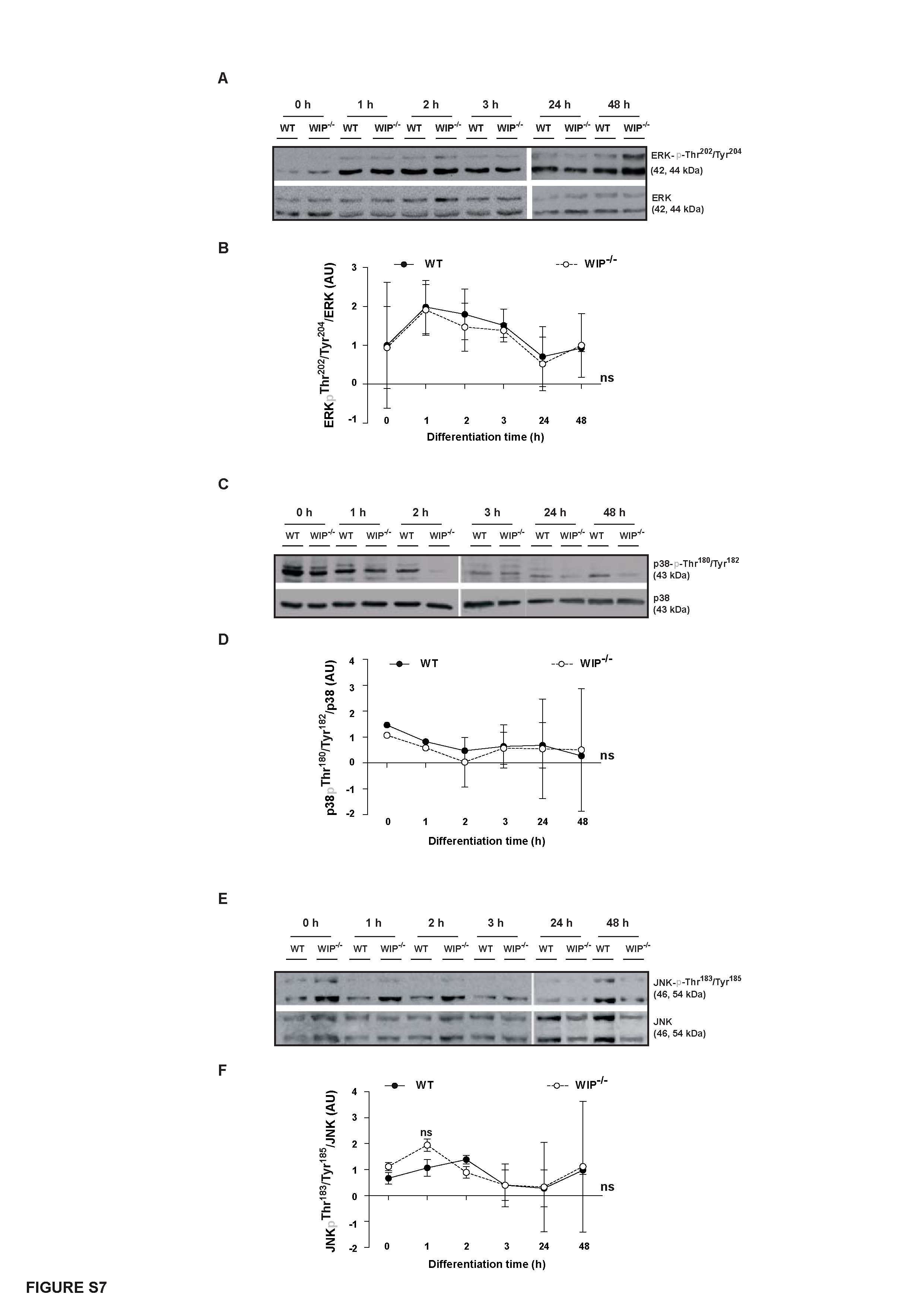

Supplement: Supplementary file 7 — Figure S7. ERK, p38, or JNK phosphorylation levels are similar in cultured WT and IP−/− neurons in the first 48 h post plating. [file BRB3-5-e00359-s007.tif]
